# Coronary sinus aneurysm associated with multiple venous anomalies

**DOI:** 10.1186/s12872-017-0532-3

**Published:** 2017-04-05

**Authors:** Guang Song, Ming Du, Weidong Ren, Ke Zhou, Lu Sun

**Affiliations:** 1 Department of Ultrasound, Shengjing Hospital of China Medical University, Shenyang, China; 2 Department of Radiology, Shengjing Hospital of China Medical University, Shenyang, China; 3 Department of Cardiac Surgery, Shengjing Hospital of China Medical University, Shenyang, China

**Keywords:** Interruption, Left inferior vena cava, Persistent left superior vena cava, Hepatic vein, Hemiazygos vein, Coronary sinus, Aneurysm

## Abstract

**Background:**

Congenital anomalies of the venous system are rare, involve the inferior vena cava (IVC), a persistent left superior vena cava (PLSVC), and the left hepatic vein (LHV), and can make cardiac diagnostic and therapeutic procedures difficult.

**Case presentation:**

We present a 67-year-old woman without heterotaxy syndrome associated with interruption of the left IVC that continued with the hemiazygos vein system, a PLSVC, and an anomalous LHV draining the into coronary sinus (CS). The venous anomalies caused a CS aneurysm. The anomalies were demonstrated by echocardiography and the diagnosis was established by contrast-enhanced computed tomography. Three days later, a coronary artery bypass graft was performed, which confirmed the diagnosis. Half a month after surgery, the pain had been relieved and the patient was discharged from the hospital.

**Conclusion:**

Echocardiography is a useful modality to diagnose and assess anomalies of the CS, including CS aneurysms. Congenital anomalies of the venous system in this case were all due to embryonic development abnormalities. Contrast-enhanced computed tomography provides a more comprehensive view of the entire course of abnormal veins.

**Electronic supplementary material:**

The online version of this article (doi:10.1186/s12872-017-0532-3) contains supplementary material, which is available to authorized users.

## Background

Congenital anomalies of the inferior vena cava (IVC) are rare, with a prevalence of 1%, and include interruption of the IVC, left IVC, and double IVC. Interruption of the IVC is a well-recognized, but uncommon anatomic anomaly [1]. The most common form of this anomaly is a right IVC that continues with the azygos vein via the right superior vena cava (R-SVC) into the right atrium (RA). A left IVC is also rare. When a left IVC is interrupted, the condition is more complicated because there are numerous routes for the return of blood [2]. In addition, interruption of the IVC is often associated with heterotaxy syndrome. If a patient does not have other features of the heterotaxy syndrome, it is easy for the examiner to miss the diagnosis. Herein, we present a patient without heterotaxy syndrome associated with interruption of the left IVC that continued with the hemiazygos vein system, a persistent left superior vena cava (PLSVC), and an anomalous left hepatic vein (LHV) connected with the coronary sinus (CS). Together, the venous anomalies caused a coronary sinus aneurysm (CSA).

## Case presentation

**Fig. 1:**
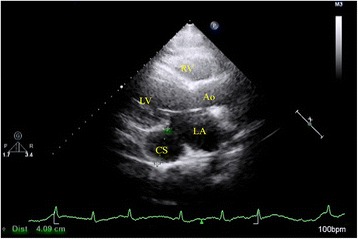
Parasternal long-axis view from echocardiography demonstrated the coronary sinus aneurysm with a diameter of 4.09 cm. Ao: aorta; CS: coronary sinus; LA: left atrium; LV: left ventricle; RV: right ventricle

**Fig. 2:**
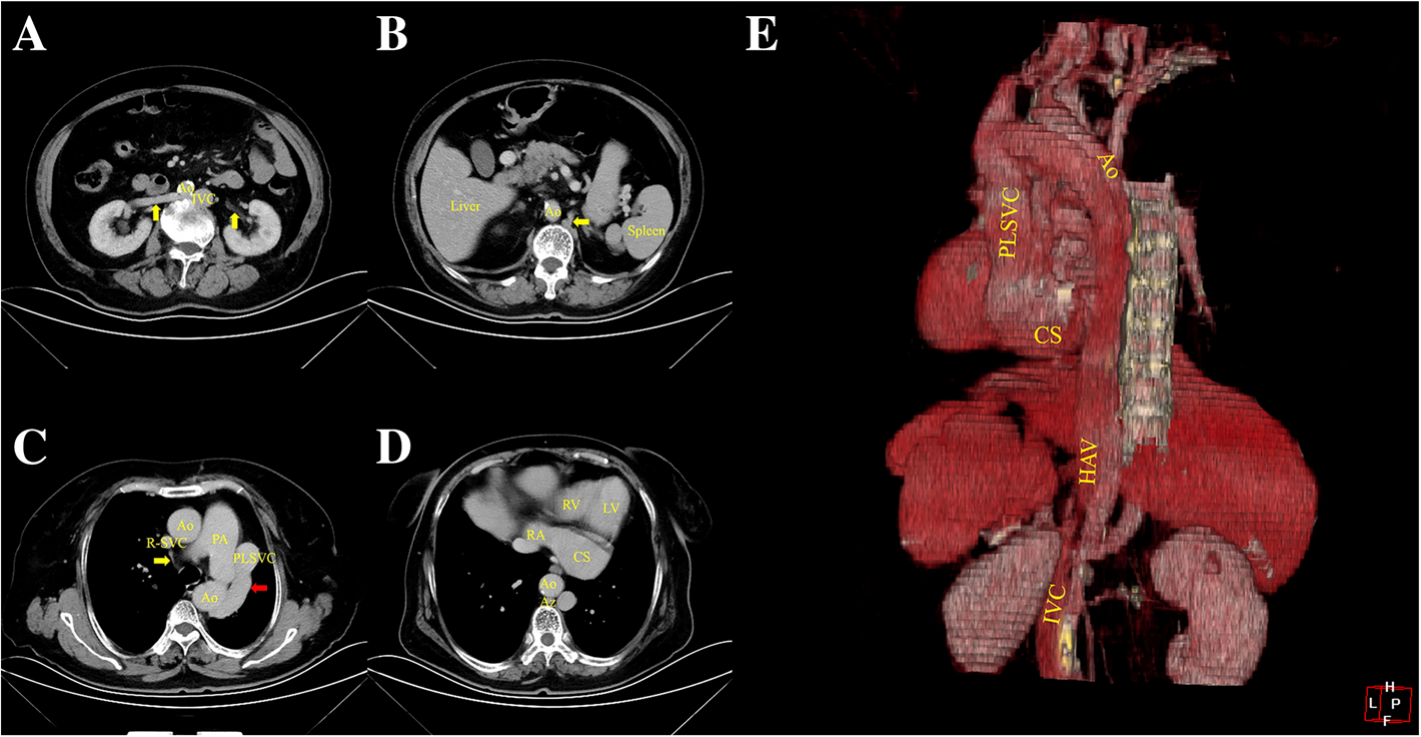
Thoracic and abdominal enhanced computed tomography demonstrating (**a**) A left IVC posterolateral to the abdominal aorta, converged by two renal veins (yellow arrow). **b** The left IVC gradually becomes thin, and continues with an enlarged hemiazygos vein (yellow arrow). Visceral position is normal. **c** The tiny azygos vein can be seen anterior to the vertebra, then crosses over the aorta and drains into the R-SVC (yellow arrow). The hemiazygos vein continues as the left superior intercostal vein via the accessory hemiazygos vein. The left superior intercostal vein eventually drained into the PLSVC at the level of the pulmonary artery bifurcation (red arrow). **d** In the left atrioventricular groove, a huge CS connected with the RA. **e** Reconstructive 3D imaging gives a better view of this anomaly. Ao: aorta; Az: azygos vein; CS: coronary sinus; HAV: hemiazygos vein; IVC: inferior vena cava; LV: left ventricle; PA: pulmonary artery; PLSVC: persistent left superior vena cava; RA: right atrium; R-SVC: right superior vena cava; RV: right ventricle

**Fig. 3:**
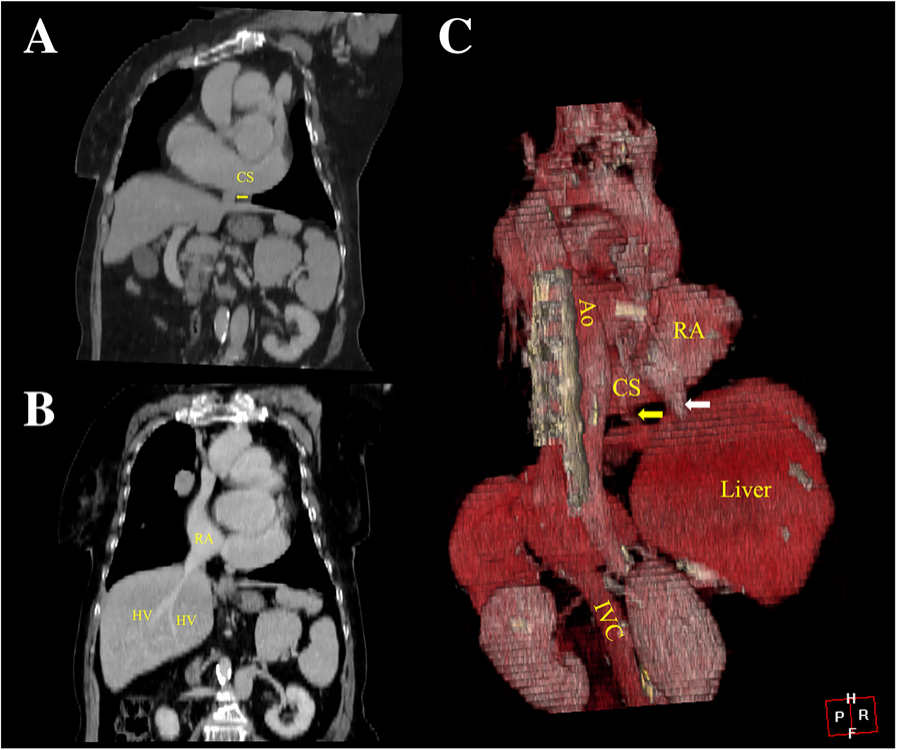
Thoracic and abdominal enhanced computed tomography demonstrating (**a**) The left hepatic vein drains into the CS (yellow arrow). **b** Other hepatic veins drain directly into the right atrium. **c** Reconstructive 3D imaging revealed that the left hepatic vein drains into the CS (yellow arrow), and other hepatic veins drain directly into the right atrium (white arrow). Ao: aorta; CS: coronary sinus; HV: hepatic vein; IVC: inferior vena cava; LHV: left hepatic vein; PLSVC: persistent left superior vena cava; RA: right atrium



A 67-year-old woman had intermittent chest pain for 1 year. She underwent coronary angiography and was diagnosed with significant coronary artery disease involving three main coronary arteries at the referring hospital. All three main coronary arteries had stenotic changes. She presented to our hospital for a coronary artery bypass graft. At the time of the clinical examination, the pulse rate was 85 beats/min and the blood pressure was 132/84 mmHg. No murmurs were auscultated. The electrocardiogram showed persistent atrial fibrillation with abnormal ST segment changes. Echocardiography demonstrated a dilated CS (Fig. 1). Thoracic and abdominal contrast-enhanced computed tomography was performed on a 64-detector row scanner (Siemens, Forchheim, Germany). Images were obtained during patient breath-holding using the following acquisition parameters: 120 kV; 240 mA; and 1.5-mm thick contiguous section. The patient received 80 mL of contrast media (Iohexol 350; GE Healthcare, Shanghai, China) using a power injector at 3.5 mL/s, and the time delays from injection of the contrast agent to scanning were approximately 20 s and 60 s for the arterial and venous phases, respectively. Thoracic and abdominal contrast-enhanced computed tomography revealed interruption of the left IVC that continued with the hemiazygos vein system, a PLSVC, and an anomalous LHV draining into the CSA (Figs. 2 & 3; Additional file 1: Movie 1). Considering her advanced age, the physicians did not plan to correct the venous anomalies. Three days later, a coronary artery bypass graft was performed, which confirmed the diagnosis. Half a month after the surgery, the pain was relieved and the patient was discharged from the hospital.

**Additional file 1: Movie 1.** Reconstructive 3D computed tomography demonstrating association with interruption of the left inferior vena cava that continues with the hemiazygos vein system, a persistent left superior vena cava, and an anomalous left hepatic vein draining into the coronary sinus. (AVI 12,902.4 kb)


## Discussion

Various diagnostic and therapeutic procedures involving the right side of the heart, such as electrophysiologic studies, right heart catheterization, cardiopulmonary bypass surgery, IVC filter placement, and temporary pacing, have increased the need for ready access to the IVC via the transfemoral route. Anatomic anomalies of the IVC can make these procedures difficult.

The interruption of the IVC has a prevalence of 0.6%–2.0% in patients with congenital heart disease and 0.3% in healthy people [3]. Embryologically, the normal IVC consists of five segments (hepatic, suprarenal, renal, infrarenal [subrenal], and iliac), which are mainly derived from the right vitelline vein (hepatocardiac canal), the right subcardinal vein, the subcardial and supracardial anastomosis, the supracardial vein, and the persistent posterior cardinal veins. Suprarenal interruption of the IVC in the current case was due to a failed connection between the right vitelline and right subcardinal veins. The blood circulating in the caudal segments of the IVC reaches the azygos/hemiazygos system by way of a persistent right/left supracardinal vein. The interruption of the IVC can be associated with cardiac abnormalities, heterotaxy syndrome (polysplenia and asplenia), deep vein thrombosis, and renal vein anomalies, and has also been reported as an asymptomatic incidental finding. Of patients associated with interruption of the IVC without other features of the heterotaxy syndrome, 62.7% do not have any cardiac malformations, which easily lead to misdiagnosis of this anomaly during echocardiography [4].

**Fig. 4:**
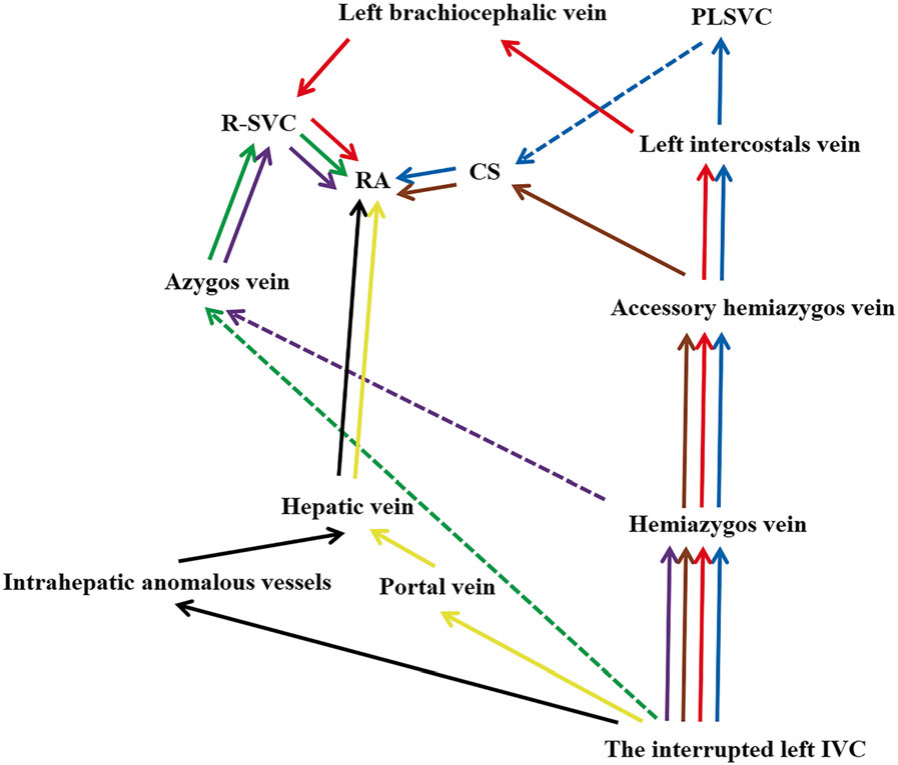
Previously described return routes for the interruption of the left IVC in the literature [2, 6–8]. The dashed and full lines represent the vessels at different positions. The dashed lines represent the relative dorsal vessels, cross behind the full lines (relative ventral vessels). CS: coronary sinus; IVC: inferior vena cava; PLSVC: persistent left superior vena cava; RA: right atrium; R-SVC: right superior vena cava

The left IVC has a prevalence of 0.2%–0.5%, and develops as a result of the persistence of the left supracardinal vein and regression of the right supracardinal vein [5]. Usually, the left IVC crosses over to the right side at the level of the renal veins without interruption. Interruption of the left IVC is rare, and only accounts for 15.5% of all interruptions involving the IVC [4]. Unlike the interrupted right IVC, which always continues via the azygos vein draining into the R-SVC (97.7%), the interrupted left IVC continues via a number of routes. Haswell et al. [2] has described three routes for the interrupted left IVC: (1) interrupted left IVC → hemiazygos vein → azygos vein → R-SVC → RA; (2) interrupted left IVC → hemiazygos vein → accessory hemiazygos vein → left intercostals vein → PLSVC → CS → RA; and (3) interrupted left IVC → hemiazygos vein → accessory hemiazygos vein → left intercostals vein → left brachiocephalic vein → R-SVC → RA. Subsequently, other routes have been discovered. Based on a review of the literature, five other types of interrupted left IVCs have been described, including the mixed type (Fig. 4) [6–8].

An anomalous LHV connection with the CS has rarely been reported in the literature; indeed, only 14 cases have been reported up to 2015 (Table 1). An anomalous LHV connection with the CS is due to the persistence of the left vitelline connection with the left sinus horn. The diagnosis of this anomaly has important clinical significance. First, an anomalous LHV connection with the CS can potentially lead to technical difficulties during trans-coronary sinus interventions and cannulation of the coronary veins for certain procedures, such as biventricular pacing. Second, this anomaly should be detected before cardiac surgery which uses a heart-lung machine because control of venous return will be challenging. Third, this anomaly should be awared in hepatic surgery and transplantation.

**Table 1:** Summary of literature involving with anomalous LHV connection with CS

No.	First Author	Year	Sex, age	First modality for diagnosis	Associated cardiovascular anomalies
1	Nabarro D	1903	M, 3 m	Autopsy	PLSVC
2	Winter FS	1954	/, /	/	PLSVC
3			/, /	/	PLSVC
4			/, /	/	PLSVC
5	van der Horst RL	1971	M, 2y	Angiography	PLSVC, ASD, PS
6	Bunger PC	1981	F, 90y	Autopsy	PLSVC, atrial fibrillation
7	Bunger PC	1982	M, 74y	Autopsy	None
8	Sanders SP	1984	/, 1d	Echocardiography	PDA, ASD, ductus venosus to the CS
9	Mantri RR	1994	F, 12y	Angiography	PLSVC, PS, bifurcation of IVC
10	Yoshinaga K	1997	M, 60y	Autopsy	None
11	Vuran C	2011	M, 6y	Intra-operation	ASD, VSD, PDA, pulmonary mass
12	Buehler M	2011	F, 19y	CT	PLSVC, subaortic valve stenosis, absence of R-SVC
13	Lee C	2013	F, 61y	CT	None
14	Morshuis WG	2015	F, 76y	Intra-operation	Three-vessel coronary artery disease

*ASD* atrial septal defect, *CSA* coronary sinus aneurysm, *CT* computerized tomography, *IVC* inferior vena cava, *PAPVC* partial anomalous pulmonary venous connection, *PDA* patent ductus arteriosus, *PLSVC* persistent left superior vena cava, *PS* pulmonary stenosis, *R-SVC* right superior vena cava, *UCS* unroofed coronary sinus, *VSD* interventricular septal defect

In patients with interruption of the IVC, the hepatic veins usually drain directly into the RA. Sometimes, the hepatic veins may connect with a residual IVC that eventually drains into the RA, pulmonary vein, or left atrium [9–11]. Our case is the first report involving multiple systemic vein anomalies.

The above-mentioned multiple venous anomalies involve the vast majority of venous blood draining into the CS. Thus, intimal hyperplasia and loss of smooth muscle and elastic tissue with replacement by connective tissue with age may aggravate the formation of CSA [12]. CSA should be diagnosed as early as possible because the CSA in children may compress the mitral annulus and limit atrioventricular flow into the left heart, eventually causing left ventricle, aortic hypoplasia, and obstructive lesions in the left ventricle [13].

## Conclusions

This is the first report involving these three co-existing systemic vein anomalies. Dilated CS by echocardiography is an important diagnostic clue for systemic anomalous venous pathways. Contrast-enhanced computed tomography provides a more comprehensive view of the entire course of abnormal veins. These congenital anomalies can be explained by the theory of embryonic development abnormalities.

## Abbreviations

CS: Coronary sinus (CS)
CSA: Coronary sinus aneurysm
IVC: Inferior vena cava
LHV: Left hepatic vein
PLSVC: Persistent left superior vena cava
RA: Right atrium
R-SVC: Right superior vena cava
